# Scanning Electron Microscopy and First Molecular Data of Two Species of *Lamproglena* (Copepoda: Lernaeidae) from *Labeo victorianus* (Cyprinidae) and *Clarias gariepinus* (Clariidae) in Kenya

**DOI:** 10.3390/pathogens12080980

**Published:** 2023-07-27

**Authors:** Nehemiah M. Rindoria, Zipporah Gichana, George N. Morara, Coret van Wyk, Willem J. Smit, Nico J. Smit, Wilmien J. Luus-Powell

**Affiliations:** 1 DSI-NRF SARChI Chair (Ecosystem Health), Department of Biodiversity, University of Limpopo, Private Bag X1106, Sovenga 0727, South Africawilmien.powell@ul.ac.za (W.J.L.-P.); 2 Department of Biological Sciences, Kisii University, P.O. Box 408, Kisii 40200, Kenya; 3 Department of Environment, Natural Resources and Aquatic Sciences, Kisii University, P.O. Box 408, Kisii 40200, Kenya; 4 Kenya Marine and Fisheries Research Institute, P.O. Box 837, Naivasha 20117, Kenya; g_morara@yahoo.com; 5 Water Research Group, Unit for Environmental Sciences and Management, North-West University, Private Bag X6001, Potchefstroom 2520, South Africanico.smit@nwu.ac.za (N.J.S.); 6 South African Institute for Aquatic Biodiversity, Makhanda 6140, South Africa

**Keywords:** freshwater fish parasites, Lake Victoria Basin, mitochondrial gene, Ningu, North African catfish, Nyando River, ribosomal gene

## Abstract

A parasitological study carried out in May 2022 and March 2023 in the Nyando River of Lake Victoria Basin, Kenya, disclosed two parasitic lernaeid copepods: *Lamproglena cleopatra* Humes, 1957, from the gills of a cyprinid, the Ningu *Labeo victorianus* Boulenger, 1901, endemic to the Lake Victoria drainage system, and *Lamproglena clariae* Fryer, 1957, from a clariid, the North African catfish *Clarias gariepinus* (Burchell, 1822). The copepods were studied and supplementary taxonomic information was presented using scanning electron micrographs and genetic data. Scanning electron microscopy (SEM) provided information on the morphology of *L. cleopatra*’s antennae, oral region, thoracic legs (2–5), and furcal rami not previously reported. Analyses of the partial fragments of 18S and 28S rDNA and cytochrome *c* oxidase subunit 1 (*cox*1) of the two parasites showed them to be distinct from all other *Lamproglena* taxa retrieved from GenBank. This study presents new taxonomic information on morphology using SEM and provides the first ribosomal (18S and 28S rDNA) and mitochondrial (mtDNA) data for these two parasite species. The *cox*1 data provided are the first for all 38 nominal species of *Lamproglena*. Notably, the study also provides a new host record for *L. cleopatra* and extends the geographical information of this species to Kenya.

## 1. Introduction

Lernaeidae Cobbold, 1879 comprises among other things the cosmopolitan parasitic freshwater copepods *Lamproglena* von Nordmann, 1832. This genus, comprising 38 nominal species, is regarded as the oldest and second-largest member of this family [1,2,3]. Out of the 38 valid species only 12 (31.59%) have been reported from Africa (*Lamproglena hemprichii* von Nordmann, 1832 (Zambia, Zimbabwe, Sudan, Egypt, Nigeria, Niger, and South Africa); *Lamproglena werneri* Zimmermann, 1923 (Sudan); *Lamproglena angusta* Wilson, 1924 (Egypt and Sudan); *Lamproglena monodi* Capart, 1944 (Malawi, Kenya, Zimbabwe, and Egypt); *Lamproglena wilsoni* Capart, 1956 (Sudan); *Lamproglena clariae* Fryer, 1956 (Malawi, Sudan, Zimbabwe, and South Africa); *Lamproglena elongata* Capart, 1956 (Sudan); *Lamproglena cleopatra* Humes, 1957 (Egypt and South Africa); *Lamproglena barbicola* Fryer, 1961; (South Africa and Kenya); *Lamproglena cornuta* Fryer, 1965 (South Sudan and South Africa); *Lamproglena hoi* Dippenaar, Luus-Powell & Roux, 2001 (South Africa); and *Lamproglena hepseti* Van As & Van As, 2007 (Botswana)). Three of the African lamproglenoids have been recorded from Lake Victoria Basin, Kenya, namely *L. barbicola*, *L. monodi,* and *L. clariae* [4,5,6,7].

Humes [8] described *L. cleopatra* from the cyprinid *Labeo forskalii* Rüppell, 1835 obtained from the Giza market in Cairo, Egypt, but this fish was presumed to have come from the Nile River in Egypt. The description of Humes [8] employed the use of light microscopy (LM) with detailed line drawings of every taxonomic structure. Six decades later, Kunutu et al. [2] gave an expanded description of this lernaeid copepod from two cyprinids from South Africa (*Labeo rosae* Steindachner, 1894 from Flag Boshielo Dam and the Leaden labeo, *Labeo molybdinus* du Plessis, 1963 from Nwanedi-Luphephe Dam) and one cyprinid Silver labeo *Labeo ruddi* Boulenger, 1907 from the River Bubye in Zimbabwe. Line drawings, scanning electron micrographs, morphometric measurements of the taxonomic features of this parasite, and a key to adult females of *Lamproglena* species were also provided [2].

Fryer [5] provided the first description of *L. clariae*, a species endemic to Africa from Mudfish *Clarias anguillaris* (Linnaeus, 1758) collected from Lake Malawi. Fryer [6,9,10] recorded the same parasite from Lake Victoria, the White Nile, Lake Albert, and Lake Malawi and provided additional taxonomic features on the number of setae on the legs and furcal rami. Thurston [11], Shötter [12], and Euler and Avenant-Oldewage [13] recorded this parasite from clariid fishes in Lake George-Edward (Uganda), the Galma River (Nigeria), and the Olifants River (South Africa), respectively. Later, Marx and Avenant-Oldewage [14] provided a comprehensive redescription of morphological features using LM and scanning electron microscopy (SEM) on specimens collected from the gills of *C. gariepinus* sampled in the Olifants River in Kruger National Park, South Africa, and the Cuando River in the Caprivi Strip, Namibia.

The present study, carried out in May 2022 and March 2023 along the Nyando River of Lake Victoria Basin in Kenya, resulted in the collection of two *Lamproglena* species, *L. cleopatra* and *L. clariae*, from the gills of the cyprinid Ningu *L. victorianus* and the clariid *C. gariepinus (*the North African catfish), respectively. The study used SEM to add new taxonomic information on the morphology of *L. cleoptra* and provided the first ribosomal DNA (18S and 28S) and mitochondrial (mtDNA) genetic data for these two parasitic copepods. The study also provided a new host record and extended the geographical report for *L. cleopatra* to Kenya.

## 2. Materials and Methods

### 2.1. Sample Collection, Examination, and Identification

In May 2022 and March 2023, 34 *L. victorianus* and 2 *C. gariepinus* were collected from the Nyando River near Ahero town [15] using an Electrofisher (SAMUS 1000, Samus Special Electronics, RX 28371, China). The fish were identified using Okeyo and Ojwang’s photographic guide [16]. The common names and nomenclature of fishes in this study followed FishBase [17].

Fish were killed by cervical dislocation [18] and gills were parasitologically examined in situ using a Leica Zoom 2000 Stereo microscope (model no. Z30V Shanghai, China). All female lernaeids found were removed using a Camel’s hair paintbrush and identified as species of *Lamproglena* using the Boxshall and Halsey [19] key. The specific species identities were determined using the Kunutu et al. [2] key. The recovered *Lamproglena* species were transferred to 70% ethanol for morphological and 96% ethanol for molecular studies. The samples were transported to the parasitology laboratory in the Department of Biodiversity, University of Limpopo, South Africa, for further examination and analysis.

### 2.2. Morphological Analyses

Five specimens preserved in 70% ethanol were prepared for LM. The specimens were cleared in lactic acid for 24 h and examined with an Olympus U-DA 0C13617 compound microscope (model BX50F no. 4C05604 Olympus Optical Co., Ltd., Tokyo, Japan) fitted with a digital camera and a drawing tube. Measurements of the body regions of the parasite were recorded (Table 1) for comparisons with previous descriptions. All measurements were expressed in millimetres (mm) unless otherwise indicated and presented as a mean with range in parentheses.

For SEM, four specimens fixed in 70% ethanol were prepared by dehydrating through graded ascending ethanol concentrations. The dehydration process consisted of 20 min sequential exchanges in increasing ethanol concentrations of 80%, 90%, 96%, 96%, 99.98%, and 99.98%. The samples were then dried for a 20 min sequential exchange using graded ascending series of Bis(trimethylsilyl)amine 30%, 40%, 50%, 60%, 70%, 80%, 90%, 100%, 100%, and 100% based on the procedures outlined by Nation [20] and Dos Santos et al. [21] with adjustments on the concentrations of ethanol and Bis(trimethylsilyl)amine and timing. Following this, the copepods were transferred into a glass desiccator for 24 h at room temperature and gold coated using a Quorum ^TM^ Q150T Emscope sputter coater (Quorum Technologies Ltd., Newhaven, U.K.). The copepods were then examined using a Zeiss Sigma 500VP scanning electron microscope (Jena, Germany) at 4 kV acceleration voltages at the University of Limpopo. Photomicrographs from LM and SEM aided in the morphometric redescription of the copepods.

### 2.3. DNA Extraction, PCR, and Sequencing

Total genomic DNA was extracted from the isolated egg strings of two *L. cleopatra* and two *L*. *clariae* specimens. This was conducted using a NucleoSpin^®^ Tissue Genomic DNA Tissue Kit (Macherey-Nagel, Düren, Germany) following the manufacturer’s instructions. Two partial fragments of the 18S rDNA and 28S rDNA genes were amplified using the primer combinations 18SF (5′–AAGGTGTGMCCTATCAACT–3′) with 18SR (5′–TTACTTCCTCTAAACGCTC–3′) and 28SF (5′–ACAACTGTGATGCCCTTAG–3′) with 28SR (5′– TGGTCCGTGTTTCAAGACG–3′). The partial fragment of the cytochrome *c* oxidase subunit 1 (*cox*1) mitochondrial gene region (mtDNA) was amplified using the primer sets LCO1490 (5′–GGTCAACAAATCATAAAGTATTGG–3′) and HCO2198 (5′ TAAACTTCAGGGTGACAAAAAATCA–3′) [22]. PCR reactions were performed in a total volume of 25 µL containing 1.25 µL of each primer (10 µM), 7 µL of molecular-grade water, 12.5 µL of DreamTaqTM Hot Start Green PCR Master Mix (2X) (ThermoFisher Scientific, Waltham, Massachusetts, USA), and 3 µL of the DNA template, following the thermocycler conditions described in Song et al. [23] for the 18S and 28S rDNA genes. The thermal cycling profile for *cox*1 mtDNA had an initial denaturation of 95 °C for 5 min, followed by 37 cycles of 95 °C for 30 s, 47 °C for 30 s, 72 °C for 1 min, and final extension at 72 °C for 7 min. Successful amplification products were verified using a 1% agarose gel electrophoresis and sent for purification and sequencing to Inqaba Biotechnical Industries (Pty) Ltd. (Pretoria, South Africa).

### 2.4. Phylogenetic Analyses

The novel sequence data obtained were assembled and inspected using the built-in De Novo Assembly tool in Geneious Prime v2022.2. (https://www.geneious.com). The resulting consensus sequences, 18S, 28S rDNA, and *cox*1, were subjected to a Basic Local Alignment Search Tool (BLAST, https://blast.ncbi.nlm.nih.gov/Blast.cgi, accessed on 10 July 2023) [24] to identify the closest congeners. Alignments for each gene/region fragment were constructed under the default parameters of MAFFT in Geneious and trimming of the 28S alignment was performed in trimAL v.1.2. using the “*gappyout*” parameter selection under default settings to remove gaps in the alignment [25]. There were no comparable sequences in GenBank for *Lamproglena* for the *cox*1 sequences generated in this study. The species used in the phylogenetic trees are outlined in Table 2. For all the alignments the parasitic copepod *Lernea cyprinacea* Linnaeus, 1758 was selected as the outgroup. The best fitting model selected for 18S and 28S rDNA alignments according to the Akaike Information Criterion (AIC) from jModelTest v2.1.4. [26] was the GTR + I + G (general time-reversible model with invariant sites and gamma distribution) model. Maximum Likelihood (ML) analyses were computed in phyML using ATGC Montpellier Bioinformatics Platform specifying AIC criterion, model selection, and a bootstrap value of 100 (http://www.atgc-montpellier.fr/, accessed on 10 July 2023) [27]. Bayesian Inference (BI) analyses were performed in MrBayes using the CIPRES [28] computational resource. The BI analyses were generated by implementing a data block criterion running two independent Markov Chain Monte Carlo (MCMC) chains of four chains for 1 million generations. A sampling of the MCMC chain was set at every 1000th generation and a burn-in was set to the first 25% of the sample generations. Phylogenetic trees generated were visualised in FigTree v1.4.4. [29]. The uncorrected pairwise distances (*p*-distances) were estimated in MEGA 7.0 [30] and the number of base pair differences was calculated in Geneious.

## 3. Results

A total of 20 female *L. cleopatra* occurred on the gills of 34 *Labeo victorianus.*

### 3.1. Taxonomic Summary

**Figure 1:**
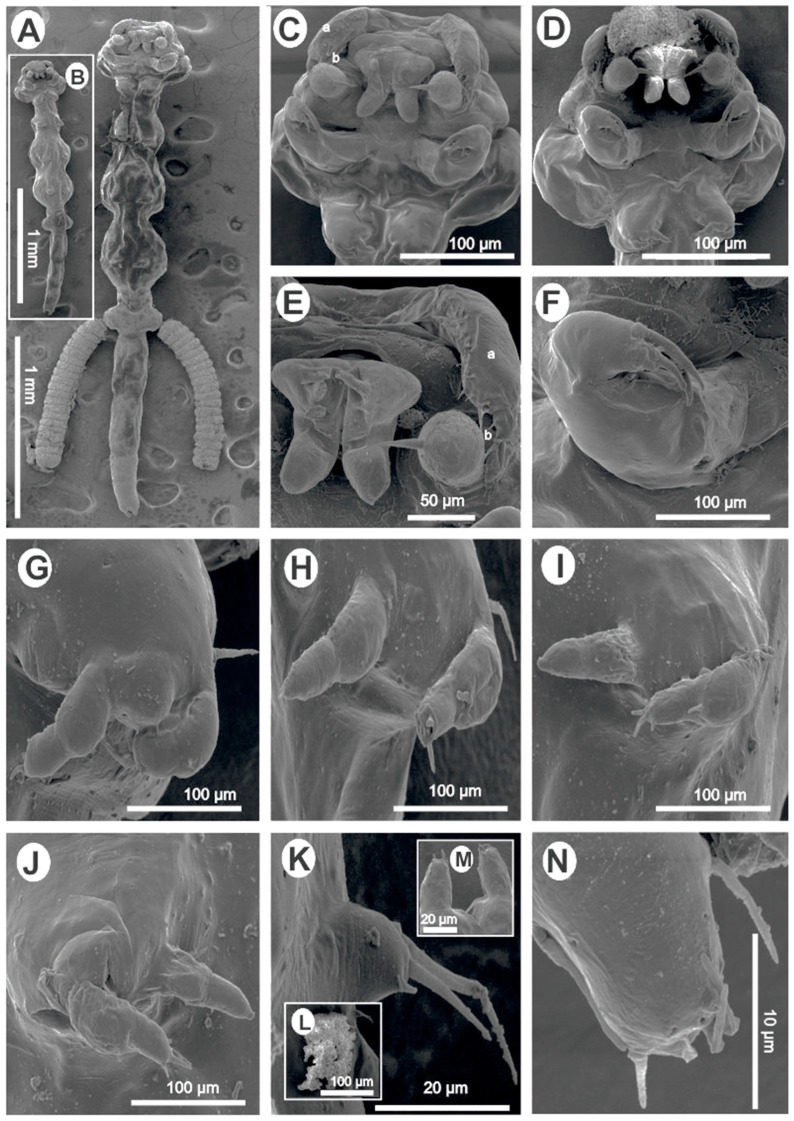
Scanning electron micrographs of a *Lamproglena cleopatra* Humes, 1957 female: (**A**,**B**) ventral view of the adult; (**C**–**E**) ventral view of cephalothorax showing antennules, antennae, oral region, and maxillae; (**F**) maxilliped; (**G**) first leg; (**H**) second leg; (**I**) third leg; (**J**) fourth leg; (**K**) fifth leg; (**L**) spermatophore; (**M**) furcal rami, showing the anal opening; (**N**) furcal rami. Abbreviations: a, antennules; b, antenna.

*Lamproglena cleopatra* Humes, 1957.
*Host*: *Labeo victorianus* Boulenger, 1901 (Cypriniformes: Cyprinidae).
*Site of infection*: Gills.
*Locality/collection date*: Nyando River-Ahero (Lake Victoria drainage system), KisumuCounty, Kenya (0°0′ 0°22′ S, 34°51′ E 35°11′ E), collected 10 May 2022 and 10 March 2023 by Drs. Nehemiah M. Rindoria and George N. Morara.
*Materials studied:* 14 specimens (5 for morphometrics, 4 for SEM, and 5 for molecular analysis).
*Deposition of voucher specimens*: A total of six voucher female specimens were deposited in the Helminthological Collection of the Institute of Parasitology, the Biology Centre of the Czech Academy of Sciences, České Budějovice, Czech Republic (IPCAS Cr-38).
*Deposition of sequences*: Sequence data obtained were deposited in GenBank: 18S rDNA (OR242501, OR242502), 28S rDNA (OR338169, OR338170), and *cox*1 (OR232207).
*Redescription* (Figure 1) *Female* (based on nine specimens, five morphometrics (all measurements in millimetres), and four SEM): Body elongated, slender, cylindrical, total length (excluding caudal rami) 2.71 (2.41–3.20) (Figure 1A,B). Body divided into cephalothorax, thorax, and abdomen (Figure 1A,B). Cephalothorax length 0.43 (0.36–0.54), width 0.56 (0.51–0.62), width represents 20.20% of total length, laterally indented; wider posterior part than thorax; U-shaped ridge on dorsal surface (Figure 1A,B). First thoracic segment fused with the head (Figure 1A–D). Second, third, and fourth thoracic segments free, with pedigerous segments distinct and well separated with indentations laterally (Figure 1A,B). Second segment 0.35 (0.24–0.42) wide, 0.26 (0.19–0.31) long. Third and fourth segments 0.42 (0.35–0.53) and 0.50 (0.37–0.54) long, respectively; width subequal 0.51 (0.39–0.59) and 0.50 (0.41–0.56), respectively, wider than the second segment (Figure 1A,B). Fifth thoracic segment narrower 0.24 (0.15–0.27), shorter 0.096 (0.07–0.13), bearing tiny fifth legs (Figure 1A,B,K). Genital segment free, wider 0.354 (0.31–0.40) than fifth thoracic segment, 0.19 (0.13–0.24) long, with egg sacs attached laterally (Figure 1A); other specimens with chitinous, kidney-shaped spermatophores attached ventrally (Figure 1A,B,L). Abdomen length 0.94 (0.79–1.10) (about 34.23% (29.43–37.69) of the total body length) composed of three approximately equal, poorly demarcated segments (Figure 1A,B). Furcal rami (Figure 1A,B,M,N) minute, 0.028 (0.02–0.03) wide, 0.037 (0.03–0.04) long. Each ramus with one long seta, one pore on inner and outer margins, and terminally with four setae, one blunt process, and two pores (Figure 1N). Antennules uniramous, indistinctly two-jointed with long swollen basal podomere bearing 11–14 naked setae and small distal podomere with 5 naked setae, 1 lateral and 4 terminal. Dorsal side of antennule with circular pores (Figure 1C–E). Antenna uniramous, indistinctly four-jointed, distal segment with five small terminal setae (Figure 1C–E). Oral region consisting of distinct projecting sucker-like with two lateral lobes from which arises two long setae and two finger-like posterior lobes (Figure 1C–E). Mandible not observed. Maxilla uniramous, rigid, covered with a thin layer through which distinct terminal spine projects, basal region finely granulated (Figure 1A–E). Maxilliped equipped with three roughly equal, curved claws, with a minute spine-like protrusion on the proximal part (Figure 1F). Legs 1–4 biramous, rami of legs indistinctly two-jointed. Endopodites of legs 1–4 all similar, terminating in a minute, rather blunt seta. Protopodite of legs 1–4 with one lateral long seta at the base before exopodite (Figure 1G–J). Exopodite of first leg first podomere with one smaller seta and four long terminal setae on the second podomere (Figure 1G). Second leg first exopodite podomere with one basal seta, second exopodite podomere with two small setae and a minute knob, an opening between setae and knob (Figure 1H). Second exopodite podomere of third and fourth legs with four setae: two long, one medium, one min (Figure 1I,J). Fifth leg made of small lobe with two long distal and one lateral seta (Figure 1K). Spermatophore observed (Figure 1I,L). Egg sac 0.98 × 0.24, containing about 20 eggs (19–22) (Figure 1A).

*Remarks:* The parasitic copepods studied here were indistinguishable from *L. cleopatra* as per the available morphological information published by Humes [8] and Kunutu et al. [2] and clearly distinct from other species of this genus. The indistinguishable features were as follows: body elongated, cylindrical and divided into a cephalothorax, thorax, and abdomen; cephalothorax broader than neck; first thoracic legs fused with the head; thoracic segments marked by lateral constrictions; indistinctly segmented abdomen; three clawed maxilliped; genital somite laterally protruding and distinctly demarcated from the rest of the thorax by a deep indent; antennule larger than antenna; biramous legs; and furcal rami with long lateral processes and terminal setae. Slight variations were noted between the present material and previous records of Humes [8] and Kunutu et al. [2], but the additional taxonomic features observed in the present material were as follows: two long setae on lateral lobes of the oral region (Figure 1C–E) and four circular pores on the furcal rami (Figure 1N).

**Figure 2:**
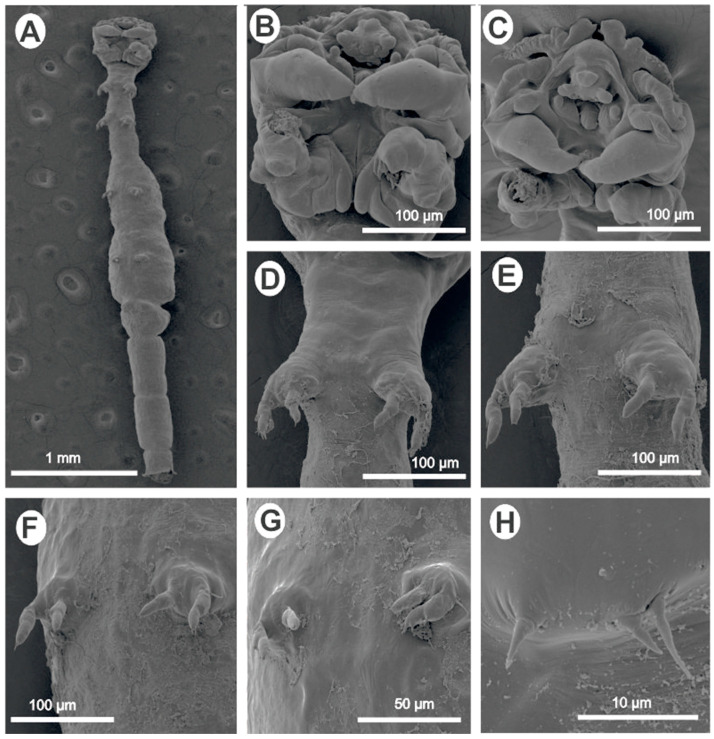
*Lamproglena clariae* Fryer, 1956 female: (**A**) ventral view of a mature adult; (**B**,**C**) ventral view of cephalothorax showing antennules, antennae, oral region, maxillae, and maxillipeds; (**D**) first leg; (**E**) second leg; (**F**) third leg; (**G**) fourth leg; (**H**) fifth leg.

*Lamproglena clariae* Fryer, 1956 (Figure 2).
*Host*: *Clarias gariepinus* (Burchell, 1822) (Siluriformes, Clariidae).
*Site of infection*: Gills.
*Locality/collection date*: Nyando River-Ahero (Lake Victoria drainage system), Kisumu County, Kenya (0°0′ 0°22′S, 34°51′E 35°11′E), collected 10 May 2022 and 10 March 2023 by Drs. Nehemiah M. Rindoria and George N. Morara.
*Materials examined:* Two specimens, one for SEM and one for molecular analysis.
*Deposition of voucher specimens*: Not deposited.
*Deposition of sequences*: Sequence data obtained were deposited in GenBank: 18S rRNA (OR242503, OR242504), 28S rRNA (OR338195, OR338196), and *cox*1 (OR232208, OR232209).
*Remarks:* Based on the morphological data available from the reports of Fryer [5] and Marx and Avenant-Oldewage [14], the present material was identical to *L. clariae*. Following a detailed redescription of this parasite using LM and SEM by Marx and Avenant-Oldewage [14], the present study only provided the SEM images to confirm the identity of our specimen and most importantly provided genetic sequences using 18S, 28S, and *cox*1 markers.

### 3.2. Molecular Identification

**Figure 3:**
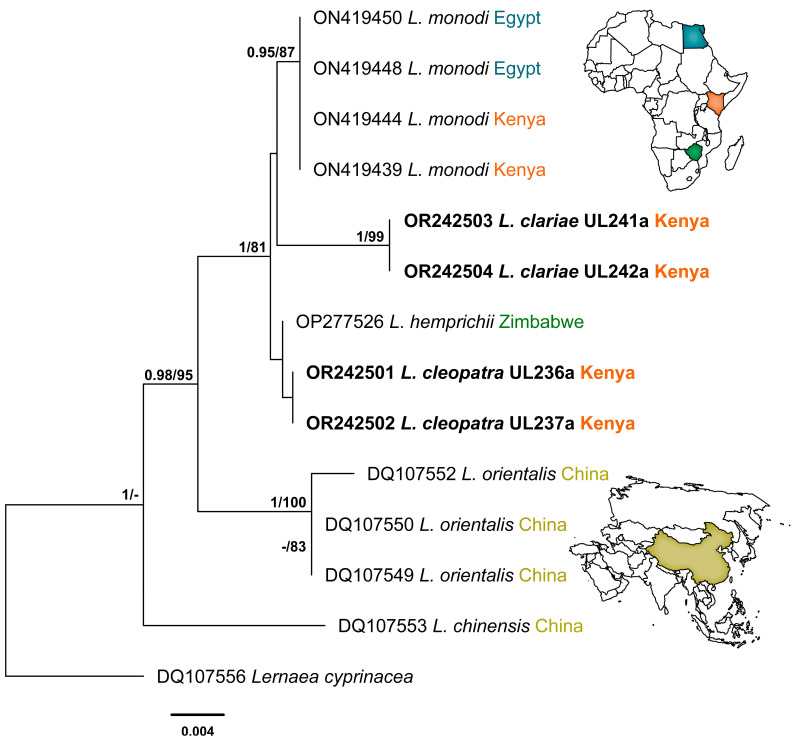
Phylogenetic relationship of *Lamproglena cleopatra* Humes, 1957 and *Lamproglena clariae* Fryer, 1956 to other Lernaeidae based on 18S rDNA. Phylogenies were reconstructed using Bayesian Inference (BI) and Maximum Likelihood (ML) with *Lernaea cyprinacea* designated as the outgroup. Sequences of the present study are highlighted in bold. Nodal support for BI and ML is indicated along the branch nodes (BI/ML); values < 0.90 (BI) and < 70 (ML) are not shown.

**Figure 4:**
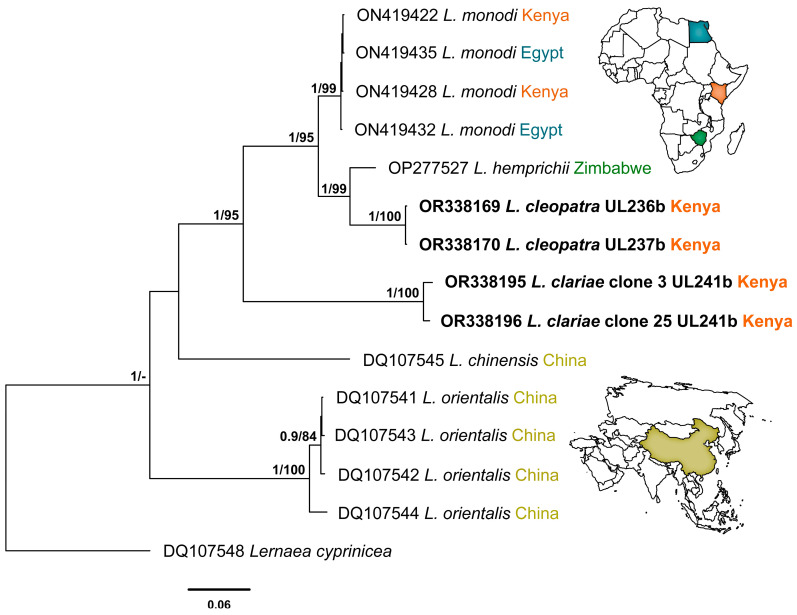
Phylogenetic relationship of *Lamproglena cleopatra* Humes, 1957 and *Lamproglena clariae* Fryer, 1956 to other Lernaeidae based on 28S rDNA. Phylogenies were reconstructed using Bayesian Inference (BI) and Maximum Likelihood (ML) with *Lernaea cyprinacea* designated as the outgroup. Sequences of the present study are highlighted in bold. Nodal support for BI and ML is indicated along the branch nodes (BI/ML); values < 0.90 (BI) and < 70 (ML) are not shown.

This study generated a total of 11 novel sequences of the three genetic markers: 5 sequences for *L*. *cleopatra* and 6 sequences for *L*. *clariae*. The Bayesian Inference and Maximum Likelihood analyses of the 18S alignment yielded similar hypotheses (nt = 1325) (Figure 3). The newly generated sequences for *L*. *clariae* and *L*. *cleopatra* fell into the clade of *Lamproglena* species previously reported from Africa with strong support. The sequences for *L*. *clariae* clustered together with high nodal support and formed a separate branch to the *L. monodi* clade with no nodal support. The novel sequences for *L*. *cleopatra* clustered together and formed a separate clade with *L. hemprichii* (OP277526) at the basal position of the African clade with no nodal support. The BI and ML analyses for the 28SrDNA dataset showed similar topologies (nt = 696) (Figure 4). A clear distinction between *Lamproglena* species from Africa and Asia clades were observed. The sequences for *L. clariae* fell at the basal position of the African clade with strong nodal support. The *L*. *cleopatra* sequences clustered with the *L. hemprichii* (OP277527) previously reported from Zimbabwe with strong nodal support.

The results from the analysis of the 18S and 28S rDNA haplotypes showed a distinct match with all sequences of the four *Lamproglena* species present in GenBank. There were no *cox*1 mtDNA sequences available in GenBank for this genus for species comparisons. The pairwise distances (*p*-distances) and number of base pair differences of *L. cleopatra* and *L. clariae* for small (18S) and large (28S) subunit rDNA and all sequences belonging to the Lernaeidae used in this analysis are presented in Table 3 and Table 4, respectively.

The two copepods in the present study, *L. clariae* and *L. cleopatra*, were distinct from other *Lamproglena* species by *p*-distances of 0.9–2.1% (13–29 bp) and 0.1–2.0% (1–30 bp) based on 18S rDNA (Table 3). For the 28S rDNA, the results showed *p*-distances of 16.8–23.7% (120–167 bp) and 7.1–23.3% (46–156 bp), respectively (Table 4). The two ribosomal DNA (18S and 28S) markers produced nearly similar topologies with insignificant intraspecific branching. The unavailability of mitochondrial (*cox*1) marker sequences in GenBank made it impossible to construct any phylogeny tree; therefore, the *p*-distance and number of base pair differences are provided for *cox*1 sequences (nt = 683) generated from the present study (Table 5).

## 4. Discussion

In the present study, lamploglenoids collected in the Nyando River, Kenya, from *L. victorianus* and *C. gariepinus* were identified as *L. cleopatra* and *L. clariae*, respectively. To a large extent, the parasites bore resemblance to the original descriptions of *L. cleopatra* by Humes [8] and *L. clariae* by Fryer [5], respectively.

For *L. cleopatra*, the original description by Humes [8] and the redescription by Kunutu et al. [2] gave illustrations with morphological and morphometric information which forms a basis for comparison with the current study. The morphometrics given in the present study (see Table 1) are within the ranges provided by Humes [8] and Kunutu et al. [2]. It is worth noting that the present study failed to compare the SEM images provided by Kunutu et al. [2] as the images provided do not conform with the original description of Humes [8] especially on the position of the first thoracic segment. The SEM images of Kunutu et al. [2] show the first thoracic segment just after the cephalothorax, which differs from the same authors’ line micrographs. The line micrographs presented by these authors are in agreement with the original description of *L. cleopatra* (see Humes [8]), which also corresponds with the morphology of the present study material (Figure 1A–D). Kunutu et al. [2] collected their study materials from three cyprinid species, *L. rosae*, *L. ruddi*, and *L. molybdinus*, from Flag Boshielo Dam, Nwanedi-Luphephe Dam, and River Bubye, respectively, the first two from South Africa and the latter from Zimbabwe, both in the Limpopo River System. We assume that the authors might have had more than one *Lamproglena* species hence the discrepancy in their line drawings and SEM images. Kunutu et al. [2] failed to provide SEM images of thoracic legs 1–4 but only provided this in the form of line micrographs, and interestingly the descriptions of the thoracic legs correspond well with the present study specimens, in which the four thoracic legs have been well illustrated (Figure 1G–J). Based on morphology, the present study recorded additional taxonomic features which were conspicuous and had not been previously recorded by Humes [8] and Kunutu et al. [2], including two long setae on lateral lobes of the oral region (Figure 1C–E) and four circular pores on the caudal region (Figure 1N).

The morphological study of the second species identified as *L. clariae* (Figure 2A–H) received little attention in the current study because Marx and Avenant-Oldewage [14] provided detailed morphological studies giving both line drawings and SEM images in addition to the original description by Fryer [5]. In this material, the present study provided an SEM image (for morphological identification) and a genetic description.

The analyses of both 18S and 28S rDNA sequence data for *L. clariae* and *L*. *cleopatra* proved to be distinct from all comparable Lernaeidae and the four *Lamproglena* sequences available in GenBank. Despite this, the pairwise distances calculated for all the *Lamproglena* species used in our analysis are from African 18S rDNA (0.9–1.0% 13 bp for *L. clariae* and 0.1–1.0% 1–14 bp for *L. cleopatra*) and 28S rDNA (1.3–18.4% 9–131 bp for *L. clariae* and 7.1–20.4% 46–135 bp for *L. cleopatra*). These pairwise distances from Africa suggest the conspecificity of *L. cleopatra* and *L. hemprichii*. Mabika et al. [31] noted that such a suggestion is improbable because of the distinctive morphology and host specificity of these two species (Cyprinidae and Alestidae, respectively). Rindoria et al. [7] found no variation in the 18S rDNA gene region for *L. monodi* collected from Egypt and Kenya, confirming the marker’s stability in distinguishing the taxa as also suggested by Mabika et al. [31]. For the mitochondrial marker (*cox*1), the present study was not able to construct any phylogeny tree due to the unavailability of sequences in GenBank for comparison. However, the study was able to give a comparison of *L. clariae* and *L. cleopatra* with *p*-distances (19.9–20.1%) and the number of base pair differences (136–137 bp) (Table 5), which confirms the distinctness of the two species.

Based on the results found in this study, the importance of global genetic data from the highly variant *cox*1 gene is highlighted, and more sequences need to be generated to help resolve the taxonomic position of all *Lamproglena* species. This study shows molecular advances in our knowledge of the diversity of *Lamproglena* and represents a significant milestone, as it is the first study to provide supplementary genetic data for *L. clariae* and *L*. *cleopatra* (the first ribosomal (18S and 28S rDNA) and the first mitochondrial (*cox*1 mtDNA) data for any of the 38 nominal species of *Lamproglena*). It also adds new taxonomic information on morphology using SEM for *L. cleopatra*. Furthermore, the study provides a new host record for *L. cleopatra* and extends the geographical information of this species to Kenya. We believe that both the morphological and molecular approaches during the classification of *Lamproglena* species are vital in expanding our understanding of their taxonomic position.

## Figures and Tables

**Table 1:** Measurements in millimetres with mean followed by standard deviation and range in parentheses of various taxonomic features of *Lamproglena cleopatra* Humes, 1957 for the present study and comparisons with previous studies.

		Humes [8]	Kunutu et al. [2]	Present Study
Country/fish species/no. measured		Egypt: *L. forskalii* n = 5	SA: *L. rosae* and *L. molybdinus* ZIM: *L. ruddi* n = 40	KEN: *L. victorianus* n = 5
Taxonomic feature				
Total length		2.60 (2.43–2.77)	2.79 ± 0,39 (1.66–3.38)	2.71 ± 0.30 (2.41–3.20)
Cephalothorax	L	0.504	-	0.43 ± 0.07 (0.36–0.54)
	W	0.375	0.58 ± 0.07 (0.41–0.71)	0.56 ± 0.05 (0.51–0.62)
Second thoracic segment	L	-	0.28 ± 0.07 (0.16–0.41)	0.26 ± 0.05 (0.19–0.31)
	W	0.291	0.32 ± 0.05 (0.19–0.40)	0.35 ± 0.07 (0.24–0.42)
Third thoracic segment	L	-	0.38 ± 0.06 (0.15–0.48)	0.42 ± 0.07 (0.35–0.53)
	W	0.422	0.43 ± 0.08 (0.20–0.59)	0.52 ± 0.08 (0.39–0.59)
Fourth thoracic segment	L	-	0.41 ± 0.07 (0.16–0.51)	0.50 ± 0.07 (0.37–0.54
	W	0.413	0.43 ± 0.08 (0.20–0.59	0.50 ± 0.06 (0.41–0.56)
Fifth leg-bearing segment	L	-	0.09 ± 0.02 (0.06–0.14)	0.096 ± 0.02 (0.07–0.13)
	W	0.212	0.22 ± 0.03 (0.16–0.30)	0.242 ± 0.05 (0.15–0.29)
Genital segment	L	-	0.17 ± 0.03 (0.13–0.22)	0.194 ± 0.04 (0.13–0.24)
	W	0.343	0.35 ± 0.06 (0.16–0.43)	0.354 ± 0.02 (0.31–0.40)
Egg sac	L	1.32	1.22 ± 0.23 (0.92–1.46)	0.976 (n = 1)
	W	0.171	-	0.24 (n = 1)
Abdomen	L	0.975	0.96 ± 0.16 (0.56–1.22)	0.94 ± 0.13 (0.79–1.10)
	W	-	0.19 ± 0.02 (0.14–0.25)	-
% of the abdomen to total body length		37	34	34
Furcal rami	L	0.039	0.04 ± 0.01 (0.03–0.06)	0.037 (0.03–0.04)
	W	0.026		0.028 (0.02–0.03)

Abbreviations: SA, South Africa; KEN, Kenya; ZIM, Zimbabwe; -, not reported.

**Table 2:** Information for the species, hosts, families, geographical localities, and accession numbers of 18S, 28S, and *cox*1 used from Lernaeidae used in molecular analyses.

Species	Host	Family	Locality	18S	28S	*cox*1	Reference
*Lamproglena orientalis*	*Squaliobarbus curriculus*	Xenocyprididae	Dangjiangkou Reservoir, China	DQ107552	DQ107544	―	Song et al. [2]
*Lamproglena orientalis*	*Chanodichthys erythropterus*	Xenocyprididae	Tangxun Lake, China	DQ107551	DQ107541		Song et al. [2]
*Lamproglena orientalis*	*Chanodichthys mongolicus*	Xenocyprididae	E-zhou farm, China	DQ107550	DQ107543	―	Song et al. [2]
*Lamproglena orientalis*	*Chanodichthys dabryi*	Xenocyprididae	Tangxun Lake, China	DQ107549	DQ107542	―	Song et al. [2]
*Lamproglena hemprichii*	*Hydrocynus vittatus*	Alestidae	Lake Kariba, Zimbabwe	OP277526	OP277527	―	Mabika et al. [28]
*Lamproglena cleopatra* Isolate UL236	*Labeo victorianus*	Cyprinidae	Nyando River, Kenya	OR242501	OR338169	―	Present study
*Lamproglena cleopatra* Isolate UL237	*Labeo victorianus*	Cyprinidae	Nyando River, Kenya	OR242502	OR338170	OR232207	Present study
*Lamproglena clariae* Isolate UL241	*Clarias gariepinus*	Clariidae	Nyando River, Kenya	OR242503	OR338195 OR338196	OR232208	Present study
*Lamproglena clariae* Isolate UL242	*Clarias gariepinus*	Clariidae	Nyando River, Kenya	OR242504	―	OR232209	Present study
*Lamproglena monodi*	*Oreochromis niloticus*	Cichlidae	Kibos Fish Farm, Kenya	ON419439	ON419422	―	Rindoria et al. [7]
*Lamproglena monodi*	*Oreochromis niloticus*	Cichlidae	Kibos Fish Farm, Kenya	ON419444	ON419428	―	Rindoria et al. [7]
*Lamproglena monodi*	*Oreochromis niloticus*	Cichlidae	Sharqia, Egypt	ON419450	ON419435	―	Rindoria et al. [7]
*Lamproglena monodi*	*Oreochromis niloticus*	Cichlidae	El-Minia, Egypt	ON419448	ON419432	―	Rindoria et al. [7]
*Lamproglena chinensis*	*Channa argus*	Channidae	Dangjiangkou Reservoir	DQ107553	DQ107545	―	Song et al. [2]
*Lernea cyprinacea*	*Chanodichthys erythropterus*	Xenocyprididae	Lake Dongxi, China	DQ107556	DQ107548	―	Song et al. [2]

― not available.

**Table 3:** Pairwise distances (%, unshaded diagonal) and the number of base pair differences (shaded diagonal) between *Lamproglena cleopatra* Humes, 1957, *Lamproglena clariae* Fryer, 1957, other *Lamproglena* species, and *Lernaea cyprinacea* Linnaeus, 1758 based on 18S rDNA (present study species % and base pairs are in bold).

		Accession Number	1	2	3	4	5	6	7	8	9	10	11	12	13	14
**1**	***L. cleopatra* UL236**	**OR242501**		**0.0**	**1.0**	**1.0**	**0.1**	**0.2**	**0.2**	**0.2**	**0.2**	**1.2**	**1.2**	**1.4**	**2.0**	**2.3**
**2**	***L. cleopatra* UL237**	**OR242502**	**0**		**1.0**	**1.0**	**0.1**	**0.2**	**0.2**	**0.2**	**0.2**	**1.2**	**1.2**	**1.4**	**2.0**	**2.3**
**3**	***L. clariae* UL241**	**OR242503**	**14**	**14**		**0.0**	**0.9**	**0.9**	**0.9**	**0.9**	**0.9**	**1.7**	**1.7**	**1.9**	**2.0**	**2.4**
**4**	***L. clariae* UL242**	**OR242504**	**14**	**14**	**0**		**1.0**	**1.0**	**1.0**	**1.0**	**1.0**	**1.9**	**1.9**	**1.9**	**2.1**	**2.6**
**5**	*L. hemprichii*	OP277526	**1**	**1**	**13**	**13**		0.3	0.3	0.3	0.3	1.1	1.1	1.4	2.0	2.4
**6**	*L. monodi*	ON419439	**3**	**3**	**13**	**13**	4		0.0	0.0	0.0	1.3	1.3	1.5	2.0	2.4
**7**	*L. monodi*	ON419444	**3**	**3**	**13**	**13**	4	0		0.0	0.0	1.3	1.3	1.5	2.0	2.4
**8**	*L. monodi*	ON419448	**3**	**3**	**13**	**13**	4	0	0		0.0	1.3	1.3	1.5	2.0	2.4
**9**	*L. monodi*	ON419450	**3**	**3**	**13**	**13**	4	0	0	0		1.3	1.3	1.5	2.0	2.4
**10**	*L*. *orientalis*	DQ107549	**16**	**16**	**24**	**24**	15	17	17	17	17		0.0	0.3	2.2	2.6
**11**	*L*. *orientalis*	DQ107550	**16**	**16**	**24**	**24**	15	17	17	17	17	0		0.3	2.2	2.6
**12**	*L. orientalis*	DQ107552	**19**	**19**	**26**	**25**	19	20	20	20	20	4	4		2.4	2.8
**13**	*L*. *chinensis*	DQ107553	**30**	**30**	**29**	**28**	29	29	29	29	29	32	32	35		2.5
**14**	*Lernaea cyprinacea*	DQ107556	**33**	**33**	**35**	**35**	34	34	34	34	34	37	37	39	38	

**Table 4:** Pairwise distances (%, unshaded diagonal) and the number of base pair differences (shaded diagonal) between *Lamproglena cleopatra* Humes, 1957, *Lamproglena clariae* Fryer, 1957, other *Lamproglena* species, and *Lernaea cyprinacea* Linnaeus, 1758 based on 28S rDNA (present study species % and base pairs are in bold).

		Accession Number	1	2	3	4	5	6	7	8	9	10	11	12	13	14	15
**1**	***L. cleopatra* UL236**	**OR338169**		**0.0**	**19.2**	**19.4**	**7.7**	**9.9**	**9.9**	**9.9**	**10.1**	**20.8**	**21.0**	**21.2**	**20.9**	**21.7**	**22.2**
**2**	***L. cleopatra* UL237**	**OR338170**	**0**		**20.4**	**20.4**	**7.1**	**9.1**	**9.1**	**9.1**	**9.2**	**22.4**	**22.6**	**22.7**	**22.5**	**23.3**	**23.0**
**3**	***L. clariae* UL241 c3**	**OR338195**	**115**	**135**		**1.3**	**18.4**	**16.8**	**16.8**	**16.8**	**16.9**	**23.5**	**23.7**	**23.5**	**23.3**	**21.1**	**24.0**
**4**	***L. clariae* UL242 c25**	**OR338196**	**116**	**135**	**9**		**17.9**	**16.8**	**16.8**	**16.8**	**16.9**	**23.2**	**23.4**	**23.2**	**23.0**	**20.7**	**24.0**
**5**	*L. hemprichii*	OP277527	**46**	**47**	**131**	**128**		6.6	6.6	6.6	6.8	19.9	20.0	20.1	19.9	19.9	22.5
**6**	*L. monodi*	ON419422	**59**	**60**	**120**	**120**	48		0.0	0.0	0.0	18.7	18.8	19.0	18.9	19.4	22.4
**7**	*L. monodi*	ON419428	**59**	**60**	**120**	**120**	48	0		0.0	0.0	18.7	18.8	19.0	18.9	19.4	22.4
**8**	*L. monodi*	ON419432	**59**	**60**	**120**	**120**	48	0	0		0.0	18.7	18.8	19.0	18.9	19.4	22.4
**9**	*L. monodi*	ON419435	**59**	**60**	**120**	**120**	48	1	1	1		18.7	18.8	19.0	18.9	19.4	22.5
**10**	*L. orientalis*	DQ107541	**122**	**146**	**166**	**164**	139	131	131	131	130		0.1	0.3	2.5	21.0	22.2
**11**	*L. orientalis*	DQ107543	**123**	**147**	**167**	**165**	140	132	132	132	131	1		0.4	2.6	21.2	22.4
**12**	*L. orientalis*	DQ107542	**124**	**148**	**166**	**164**	141	133	133	133	132	2	3		2.7	21.3	22.5
**13**	*L. orientalis*	DQ107544	**125**	**149**	**167**	**165**	142	135	135	135	134	20	21	22		20.7	22.0
**14**	*L. chinensis*	DQ107545	**132**	**156**	**154**	**151**	144	141	141	141	140	151	152	153	151		22.7
**15**	*Lernaea cyprinacea*	DQ107548	**155**	**176**	**195**	**195**	182	183	183	183	183	181	182	183	182	180	

**Table 5:** Pairwise distances (%, unshaded diagonal) and the number of base pair differences (shaded diagonal) between *Lamproglena cleopatra* Humes, 1957, *Lamproglena clariae* Fryer, 1957, other *Lamproglena* species, and *Lernaea cyprinacea* Linnaeus, 1758 based on *cox*1 (present study species % and base pairs are in bold).

	Accession Number	OR232207 *L. cleopatra*	OR232208 *L. clariae*	OR232209 *L. clariae*	NC 025239 *Lernaea cyprinacea*
***L. cleopatra* UL237**	**OR232207**		**20.1**	**19.9**	26.8
***L. clariae* UL241**	**OR232208**	**137**		**0.1**	26.2
***L. clariae* UL242**	**OR232209**	**136**	**1**		26.4
*Lernaea cyprinacea*	NC 025239	183	179	180	

## Data Availability

The data presented in this study are openly available in GenBank (accession number OR232207-OR232209; OR242501-OR242504; OR338169 and OR338170; OR338195 and OR338196) and the Helminthological Collection of the Institute of Parasitology, the Biology Centre of the Czech Academy of Sciences, České Budějovice, Czech Republic (voucher numbers IPCAS Cr-38).
